# Integrating liquid chromatography mass spectrometry into an analytical protocol for the identification of organic colorants in Japanese woodblock prints

**DOI:** 10.1038/s41598-020-77959-2

**Published:** 2020-12-01

**Authors:** Marc Vermeulen, Diego Tamburini, Emily M. K. Müller, Silvia A. Centeno, Elena Basso, Marco Leona

**Affiliations:** 1 Department of Scientific Research, The Metropolitan Museum of Art, 1000 Fifth Avenue, New York, NY 10028 USA; 2 Department of Scientific Research, The British Museum, Great Russell Street, London, WC1B 3DG UK; 3 Department of Paper Conservation, The Metropolitan Museum of Art, 1000 Fifth Avenue, New York, NY 10028 USA; 4 Present Address: Northwestern University–Art Institute of Chicago Center for Scientific Studies in the Arts (NU-ACCESS), 2145 Sheridan Road, Tech K111, Evanston, IL 60208 USA; 5 Present Address: Department of Conservation and Scientific Research, Freer Gallery of Art and Arthur M. Sackler Gallery, National Museum of Asian Art, Smithsonian Institution, 1050 Independence Ave SW, Washington, DC 20560 USA

**Keywords:** Imaging studies, Mass spectrometry, Characterization and analytical techniques, Imaging techniques, Mass spectrometry, Raman spectroscopy

## Abstract

Three Japanese woodblock prints from the Edo period (1603–1868) underwent a scientific investigation with the aim of understanding the changes in the colorants used in Japanese printing techniques. A multi-analytical approach was adopted, combining non-invasive techniques, such as fiber optic reflectance spectroscopy (FORS), Raman spectroscopy, multispectral imaging (MSI), and macro X-ray fluorescence (MA-XRF) with minimally invasive surface-enhanced Raman spectroscopy (SERS). The results enabled many of the pigments to be identified and their distribution to be studied, apart from two shades of purple of organic composition. Consequently, the potential of high-pressure liquid chromatography tandem mass spectrometry (HPLC–MS/MS) was explored for the first time with application to Japanese woodblock prints. The intrinsic sensitivity of the instrument and an effective extraction protocol allowed us to identify a mixture of dayflower (*Commelina communis*) blue and safflower (*Carthamus tinctorius*) red in purple samples constituted of 2–3 single fibers. In addition to the innovative integration of MA-XRF and HPLC–MS/MS to investigate these delicate artworks, the study concluded on the use of traditional sources of colors alongside newly introduced pigments in late Edo-period Japan. This information is extremely important for understanding the printing practices, as well as for making decisions about display, conservation, and preservation of such artworks.

## Introduction

Japanese woodblock prints are among the most popular forms of artworks on paper. The prints usually depict ordinary elements of life, such as landscapes, tales from history, scenes from the Kabuki theatre, as well as courtesans, or *geishas*. Due to their low manufacture cost, woodblock prints were mass produced and widely distributed. In the Edo period (1603–1868 AD), Japanese prints became one of the dominant art movements. The vibrant colors and extremely various palette of nuances, as well as the particularly appealing style of this form of art, have not only attracted the eyes of collectors for centuries, but also continue to capture the attention of the general museum public. Nowadays, Japanese woodblock prints can be found in great numbers in many museum collections, including the Metropolitan Museum of Art’s (New York City, USA).

In a similar way, Japanese woodblock prints have also attracted the attention of scholars and, more recently, scientists^1–13^. Driven by research questions such as the lightfastness of colors, the chronology of the use of certain pigments^11,12,14^, the introduction of new materials in Japan^4,11^, the understanding of the creation sequence of prints^4,13,15^, and the transition between the Edo period and the Meiji era^4^, researchers have developed analytical protocols with particular attention to the investigation of Japanese woodblock prints^1,4,5,14,15^. These protocols include mostly non-invasive techniques. In fact, artworks on paper are often considered too precious or too fragile to allow sampling. The risk of damaging the appearance of the object is generally too high, thus microscopic, spectroscopic and imaging techniques are mostly used. Imaging techniques are well-suited for the study of Japanese prints due to their planarity and their relatively simple pigments and colorants composition, which are often applied in one single layer and, regularly, not mixed^16^. As a result, multispectral imaging (MSI) has been applied several times to the study of Japanese woodblock prints^6,14,17–19^. Combined with spectroscopic single point analytical techniques, this approach generally produces reliable results in terms of identification of mineral pigments^4,5,14,15,17^ and, to some extent, organic colorants^14,19–21^. Nonetheless, to date, MA-XRF has never been used for the investigation of coloring materials in Japanese woodblock prints.

The scenario becomes more complicated when organic colorants are present. Some dye molecules, especially most yellow dyes, do not produce signals specific enough to enable their identification non-invasively^1,5,20,22,23^. In situ Raman spectroscopy may be useful for the identification of synthetic organic colorants, but the sensitivity and background fluorescence may be issues in the case of colorants from natural sources^24,25^. The development of surface-enhanced Raman spectroscopy (SERS) represented a breakthrough in this regard^26–30^. As far as sample size is concerned, SERS is a very compelling technique, as it only requires a very small amount of sample. For textiles or works on paper, a single 80 µm fiber is generally enough. Due to the low level of invasiveness and the minimal effect on the overall appearance of the object, such sampling is generally perceived as acceptable for works on paper, including prints. While SERS is very sensitive to detect many colorants, the technique presents some limitations, especially for yellow colorants or some complex mixtures^28,31^.

All these limitations can be theoretically overcome by using high-pressure liquid chromatography tandem mass spectrometry HPLC–MS/MS. This technique gives information on the exact molecular composition of a sample, as all the dye molecules present are chromatographically separated and identified singularly^32,33^. Mixtures are therefore easily resolved, and dye sources can be identified with extreme precision, down to the exact plant or animal species^34,35^. Unfortunately, so far, the amount of sample reported to be needed for such analysis, although very small, does not appear to be suitable to analyze Japanese woodblock prints. However, no attempt has been made to analyze a small number of single fibers peeled off from the surface of a print, in a similar way to what is done when sampling for SERS. Therefore, one of the aims of this study was to test the feasibility of such sampling and subsequent HPLC–MS/MS analysis, in order to identify the sources of the organic colorants used to obtain particular hues, such as purple shades, which may prove challenging with other techniques.

In this framework, a multi-analytical approach was used and included non-invasive methods, such as MSI, MA-XRF, single spot XRF, FORS, Raman spectroscopy, and, when deemed required, minimally invasive techniques, such as SERS and HPLC–MS/MS. This approach was used for three Edo-period Japanese woodblock prints, in order to investigate the possible combined use of traditional practice alongside newly introduced color formulations.

## Materials and methods

### Woodblock prints

**Figure 1:**
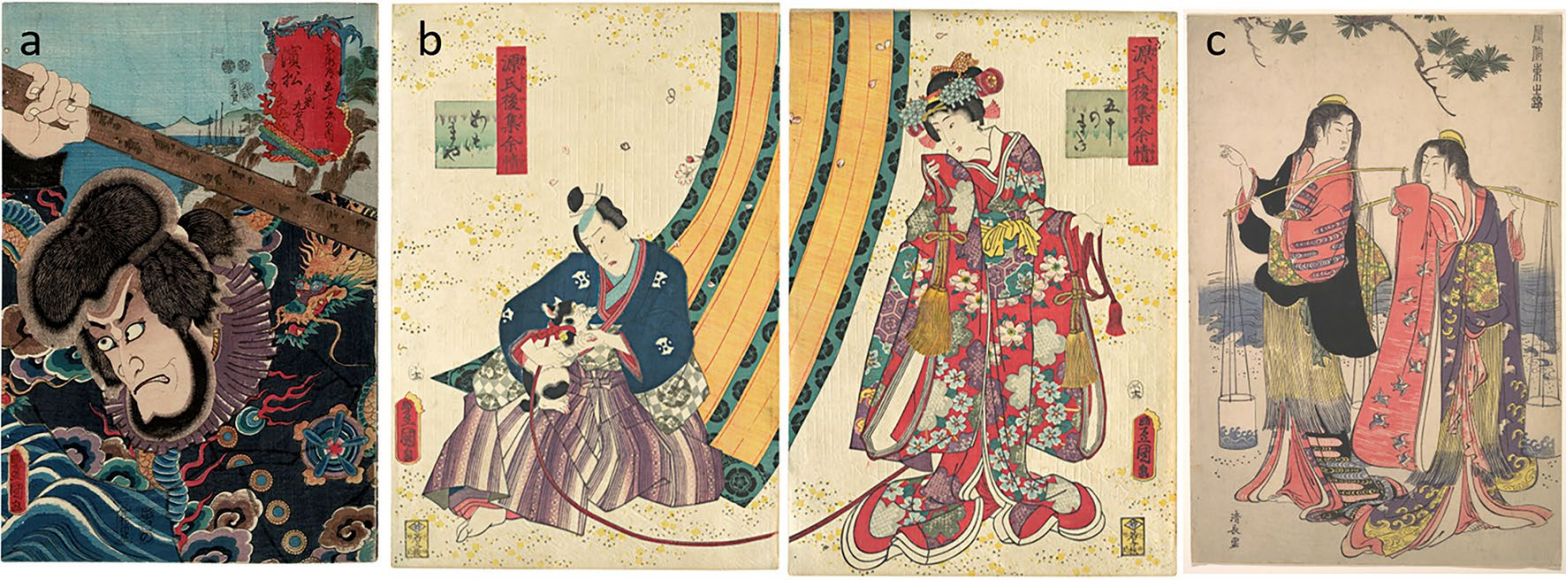
Impressions of (**a**) 15.07 h, print A, “Portrait of kabuki actor Ichikawa Ebizô V as Hamamatsu in the play Kezori Kuemon” from the series “The Fifty-Three stations of the Tokaido” by Utagawa Kunisada, 2nd month of 1852, 35.2 × 23.8 cm, Collection of Henry D. Smith II; image: The Metropolitan Museum of Art; (**b**) JP3683a,b, print B, “Parody of the Third Princess and Kashiwagi—“Chapter 50: A Hut in the Eastern Provinces” by Utagawa Kunisada, 2nd month of 1852, each 36.2 × 24.8 cm, The Metropolitan Museum of Art, Gift of Lincoln Kirstein, 1985; (**c**) JP2614, Print C, “The Salt Maidens Murusame and Matsukaze” by Torii Kiyonaga, ca. 1786, 38.4 × 25.7 cm, The Metropolitan Museum of Art, The Howard Mansfield Collection, Purchase, Rogers Fund, 1936.

Two Japanese woodblock prints (Fig. 1a,b) were scientifically investigated to determine their color palette. The purple color from a third print (Fig. 1c) was included for means of comparison with the two other prints. Two prints (accession numbers JP3683 a,b—Fig. 1b, and JP2614—Fig. 1c) belong to the Metropolitan Museum of Art, New York, whereas the third one (collection number 15.07 h—Fig. 1a) is part of the private collection of Henry D. Smith II. All three prints were produced during the Edo period (1603–1868 AD): Torii Kiyonaga’s *The Salt Maidens Murusame and Matsukaze* (JP2614, Fig. 1c) in 1786; Utagawa Kunisada’s prints *Portrait of kabuki actor Ichikawa Ebizô V as Hamamatsu in the play Kezori Kuemon* (15.07 h, Fig. 1a) and *Parody of the Third Princess and Kashiwagi: “Chapter 50: A Hut in the Eastern Provinces* (JP3683 a,b, Fig. 1b) in 1852 and 1858 respectively. To ease the reading, the woodblock prints studied here will be referred as follows: 15.07 h, print A; JP3683a,b, print B; and JP2614, print C.

The prints were examined non-invasively using MSI, MA-XRF, Raman and FORS. Nonetheless, eight micro-samples (1–8) were taken to perform organic colorant analyses using SERS and HPLC-ESI-Q-ToF. The single paper fibers (*ca.* 100 µm in length for SERS and 2–3 mm long for HPLC-ESI-Q-ToF) were sampled from the front of the prints under magnification using a tungsten needle and/or tweezers. Raised from the surface or partially detached fibers were selected to minimize the damage to the prints.

Analyses locations for FORS and Raman, as well as sampling locations for SERS and HPLC-ESI-Q-ToF, can be found in Supplementary Information (Figs. S1, S2 and Table S1).

### MSI

Multispectral imaging (MSI) was performed on prints A and B.

Successful scientific research has to follow standardized procedures in order to achieve reliable and reproducible results^36,37^. Nonetheless, no single protocol is followed by all institutions and, therefore, it is important to document used procedures. In this study MSI was performed using the Met’s protocol for examination under visible (VIS), infrared (IR) and ultraviolet (UV) radiation.

A Nikon D90 (modified, IR filter removed) and lens Nikkor 18–105 mm (minimum focus distance: 1.48″/45 cm; filter thread 67 mm) were used with an IDAS filter (UV and IR blocking filter, 67 mm; sharp cut-off and very low in-ban reflectivity to minimize reflections between the imaging sensor and filter) for VIS images. In order to capture UV-induced visible luminescence (UVL) images, a B + W 67 mm Yellow MRC 022M filter was attached onto the IDAS filter and two SuperBright II (LW-350) lamps were used as source of UV radiation. The same illumination set up was used for UV reflected (UVR) images. PECA 900 (67 mm) and X-Night BP1 (72 mm) filters were combined to extract and record wavelengths in the UV spectrum only. IR reflected (IRR) images were obtained using a PECA 910 (67 mm) filter and two Lowel ViP Pro-Light lamps.

All images were captured with the software Camera Pro 2 including the following Color charts in each shot to provide standardized results: XRite Colorchecker Passport, Spectralon Diffuse Reflectance Standards, Target UVTM. Additional steps of imaging included flat fielding and post processing of images in Photoshop lightroom (version 5 or higher) and Adobe Flat Field Plug-In.

False Color (FC) images were also obtained, as these can yield additional specific information on pigments and colorants. UVRFC and IRRFC were created. Individual color channel image data (red, green and blue channels, RGB) were transferred into a yellow/magenta/cyan system in Photoshop. UVRFC images were created by transferring the blue and green channels of the visible image in cyan and magenta respectively, whereas the yellow channel was substituted with the recolored UVR monochrome image. Comparably, IRRFC images were produced by transferring the green and red channels of the visible image into yellow and magenta respectively, while the cyan channel was substituted with the IRR image. The acquisition of all the images took approximately 1 h, of which 15 min were dedicated to UVL and UVR imaging. The risk of damage when exposing an object to a UV source for such a short amount of time can be considered negligible^20^.

Comparison of the multispectral images with the Met in-house database published in^14^ and other available databases published in^19,20^ was used in order to interpret the images obtained.

### MA-XRF

Macro X-ray fluorescence (MA-XRF) was carried out on prints A and B.

For print B, a Bruker M6 Jetstream instrument with the X-ray source operated at 50 kV and 0.5 mA was used. The full prints were mapped with a 580-µm spot size and a 650-µm step size, with a dwell time of 90 ms/pixel. All elemental maps were processed using the Bruker M6 Jetstream software^38^. Spectra were deconvoluted using this software and the elements to map were selected upon inspection of the maximum pixel spectrum and the map spectrum.

Due to unavailability of the Bruker M6 Jetstream instrument at the time of the study of print A, its MA-XRF was carried out with an XGLab Elio energy dispersive X-ray fluorescence analyzer, with a high resolution large area Silicon Drift Detector with 130 eV at manganese (Mn) Kα with 10 kcps input photon rate (high resolution mode), 170 eV at Mn Kα with 200 kcps input photon rate (fast mode). The system is equipped with changeable filters, and a rhodium (Rh) transmission target with 50 kV maximum voltage and 4 W maximum power. The size of the analyzed spot is 1 mm in diameter. Elemental 2D mapping of the surface can be achieved through automatic XY raster scanning. In our case, the following measurement conditions were used: measurement time per point 1 s, tube voltage 50 kV, tube current 80 µA, movement delay 100 ms, dimension of the scanned area 4 × 8 cm^2^ (40 row and 80 columns with 1 mm distance between 2 row/columns). The maps were acquired using the Elio software and elaborated using PyMCA software suite^39^. The area of print A to be analyzed was selected based on the presence of most of the colors within the investigated area. As far as safety of exposure of the artwork to X-ray radiation is concerned, no significant damage has ever been reported in the literature for analyses performed under similar experimental conditions, using both MA-XRF or point XRF^40^. MA-XRF experiments at synchrotron facilities explored this concern using irradiation more intense that what would be expected in practice and at no point was damage observed^41,42^.

### FORS

FORS was performed with an Ocean Optics QE65 Pro spectrometer (Dunedin, FL, USA) using the analytical protocol described in^4,14^. To summarize, the instrument was equipped with an Ocean Optics HL-2000 tungsten halogen light source. The detector and light source were connected with a high OH– fused silica fiber optic bundle consisting of eight 600 µm fibers arranged around a single 800 µm fiber. The distal end of the probe is partially fused to form a spherical lens that focuses both illumination and detection fibers to the same spot. The working distance between the probe and the prints was approximately 1 mm. The spectral range of the detector is 200–1000 nm. Nonetheless, due to poor light output on the extremes of the range, only the range between 400 and 900 nm was considered. Spectra were referenced against the SRS-99-010 Spectralon diffuse white standard provided by Labsphere (North Sutton, NH, USA) and acquired for 10 ms. Spectra were not corrected nor normalized unless otherwise indicated. Identifications of the various pigments and colorants found in the prints were based on previously published FORS reference libraries^14,19,43^. The system was managed by the OceanView 1.6.7 software for Windows and data processed with OriginLab 8.0.

### Raman spectroscopy and SERS

Micro-Raman spectra were acquired with a Bruker Senterra Raman spectrometer coupled with an LMPlanFL Olympus 50× long working distance microscope objective and a charge-coupled device (CCD) detector, as described in^4,14^. A continuous wave diode laser, emitting light at 785 nm, was used as the excitation source, and a 1200 rulings/mm holographic grating provided a spectral resolution of 3–5 cm^−1^. All Raman spectra presented in this work were recorded with 1 mW output laser power at the sample. Acquisition time varied upon the color investigated. For the blue and green areas, the acquisition time was of 4 min (four successive 60 s accumulations), while it was set to 1 min (two successive 30 s accumulations) for all other colors. The blue colorants spectra were acquired in the 1515–2635 cm^−1^ range, permitting the visualization of the characteristic bands of both indigo and Prussian blue, while the other colored particles were acquired in the 60–1500 cm^−1^ range, suitable for most inorganic pigments. Spectra were acquired using OPUS 7.0 Raman software and processed with OriginLab 8.0. Spectra were not corrected nor normalized unless otherwise indicated. The spectra obtained from the prints were compared to reference spectra collected in house and to those in published databases^44–46^.

The initial investigation of the organic colorants used in the prints was realized using SERS. The silver colloids were prepared by microwave-supported reduction of Ag_2_SO_4_ in the presence of glucose and sodium citrate^47^. The colloids were concentrated five times by centrifuging 1 ml of colloids and replacing 950 µl of the supernatant liquid with 150 µl of ultrapure water.

Samples were obtained from the prints by removing small fragments of organic colorant accumulated at the borders of the printed areas and/or fragments of single paper fibers (ca. 100 µm in length) using a tungsten needle and/or tweezers. To minimize the damage to the prints, samples (fibers or organic colorant) already raised from the surface or partially detached were selected. The samples were analyzed as sampled, as well as following hydrolysis. When hydrolysis was used, the samples were hydrolyzed prior to analysis by exposing them to HF vapor for 3 min. After the treatment, a 2 µl drop of colloids was deposited onto the sample, followed by a *ca.* 0.5 µl drop of 0.1 M KNO_3_ solution to induce aggregation.

SERS spectra were acquired with the Bruker Senterra Raman spectrometer previously described, using an LMPlanFL Olympus 20× long working distance microscope objective. A continuous wave diode laser, emitting light at 488 nm (Spectra Physics Cyan), was used as the excitation source, and a 1200 rulings/mm holographic grating provided a spectral resolution of 3–5 cm^−1^, keeping the laser power on the specimen below 0.4 mW. The laser beam was focused inside the silver nanoparticles drop. All spectra were acquired for 30 s using OPUS 7.0 Raman software and processed with OriginLab 8.0.

### HPLC-ESI-Q-ToF

The samples taken from the woodblock prints were constituted of 2–3 single fibers 2–3 mm long. These are generally considered very small samples, and this was the first attempt to obtain reliable qualitative information from such a small amount of material. The sample taken from the dayflower blue reference for data comparison was slightly larger. This reference sample was a small piece of paper (ca. 5 × 5 cm) dyed with dayflower (*Commelina communis*) purchased as such from Tanaka-Nao Dye Supplies in Kyoto, Japan. The molecular extraction was performed using a method published in^48^, which briefly consists of a double mild extraction procedure, using DMSO first, and secondly a mixture of methanol/acetone/water/0.5 M oxalic acid 30:30:40:1 (v/v/v/v). Analyses were carried out using a 1260 Infinity HPLC (Agilent Technologies), coupled to a Quadrupole-Time of Flight tandem mass spectrometer 6530 Infinity Q-ToF detector (Agilent Technologies) by a Jet Stream ESI interface (Agilent Technologies). Separation was achieved using a Zorbax Extend-C18 column (2.1 mm × 50 mm, 1.8 μm particle size) with a 0.4 mL/min flow rate and 40 °C column temperature, and a gradient of water with 0.1% formic acid (eluent A) and acetonitrile with 0.1% formic acid (eluent B). The elution gradient was programmed as follows: initial conditions 95% A, followed by a linear gradient to 100% B in 10 min, and held for 2 min. Re-equilibration time for each analysis was 10 min. 5 μL injection volume was adopted for MS experiments and 10 μL for MS/MS experiments. The ESI operating conditions were: drying gas (N_2_, purity > 98%) temperature 350 °C and 10 L/min flow; capillary voltage 4.0 kV; nebulizer gas pressure 40 psig; sheath gas (N_2_, purity > 98%) temperature 375 °C and flow 11 L/min. High resolution MS and MS/MS spectra were acquired in both negative and positive mode in the range 100–1700 *m/z*. The fragmentor was kept at 150 V, nozzle voltage 1000 V, skimmer 65 V, octapole RF 750 V. For the MS/MS experiments, different voltages (from 20 to 40 V) in the collision cell were tested for Collision Induced Dissociation (CID), in order to maximize the information obtained from the fragmentation of the single molecules. The collision gas was nitrogen (purity 99.999%). The data were collected by targeted MS/MS acquisition with an MS scan rate of 1.0 spectra/s and a MS/MS scan rate of 1.0 spectra/s. Auto-calibration was performed daily using Agilent tuning mix HP0321 (Agilent Technologies) prepared in 90% water-10% acetonitrile. The extraction method and analytical conditions have proven suitable for the analysis of organic colorants in both dyes^21,34,48–50^ and pigments formulations^50–52^.

MassHunter Workstation Software was used to carry out mass spectrometer control, data acquisition, and data analysis. In particular, extract ion chromatograms were obtained using the software EIC function and selecting the mass range corresponding to the calculated mass ± 0.001 *m/z*.

## Results and discussion

### Visual examination

Upon examination under the microscope, the prints appeared in very good condition and did not present any significant sign of fading or degradation. Some fibers appeared lifted as it would be expected due to the preparation of the prints as well as due to handling over time.

Microscopic observations of the various colored areas indicated that blue was applied as a pure color. No individual pigment particles were noticed. In the green areas, however, yellow particles were clearly observed, indicating the use of a blue and yellow mixture to yield the final hue. Red and yellow areas did not present any visible pigment particle, which may indicate the use of organic colorants. Finally, the observation of the purple areas did not present any variation in color, as it would be expected for mixtures of blue and red. This initially suggested the use of a single purple colorant.

### Imaging techniques: MA-XRF and MSI

These two techniques led to the tentative identification of pigments and colorants based on their elemental composition (MA-XRF) or their characteristic absorbance, reflectance and luminescence properties in different parts of the electromagnetic spectrum (MSI)^20,53^.

**Figure 2:**
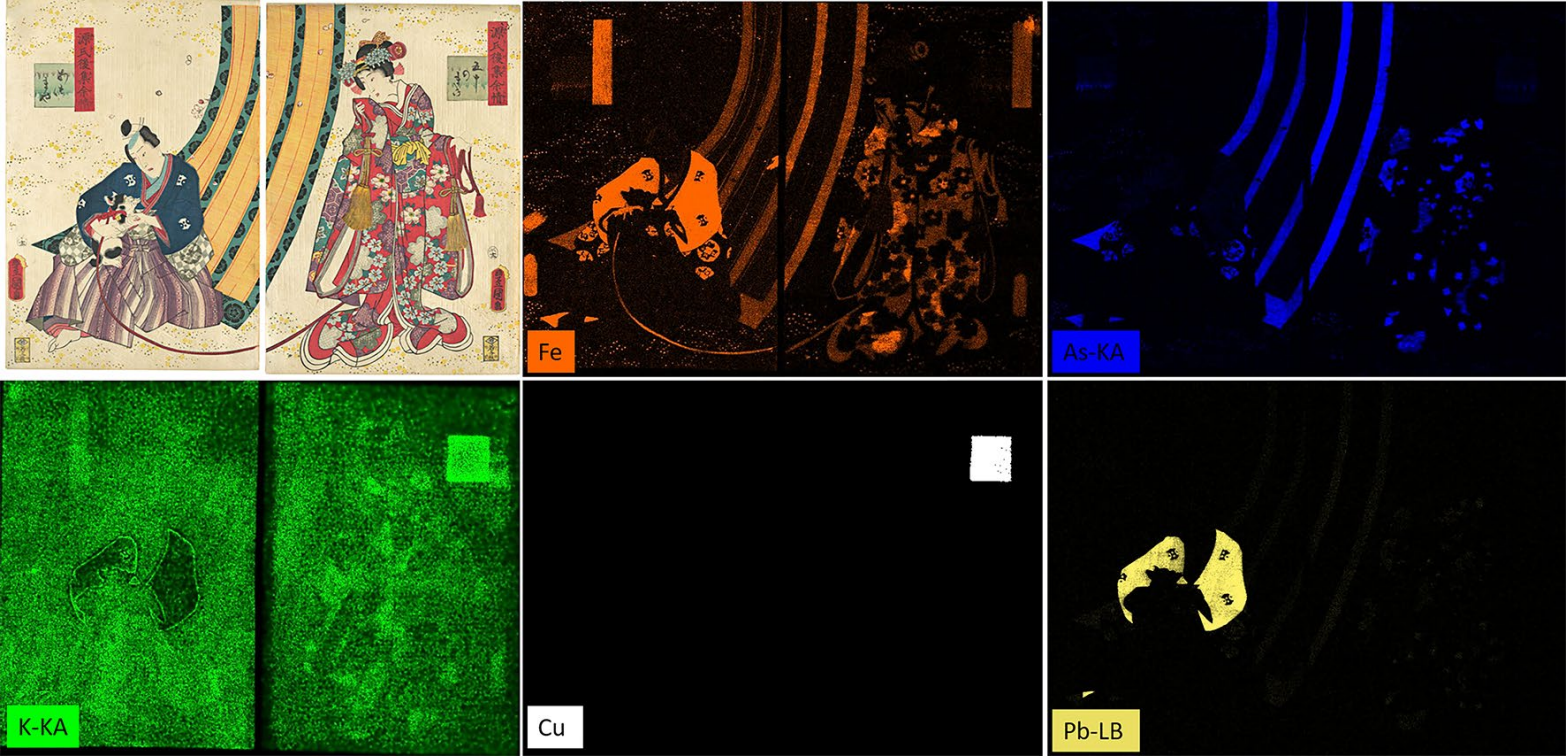
MA-XRF elemental maps for print B, showing the distribution of iron (Fe, orange), arsenic (As, blue), potassium (K, green), copper (Cu, white) and lead (Pb, yellow), as detected in the red, green and blue areas of the prints. The elemental maps were created using the Bruker M6 software package.

**Figure 3:**
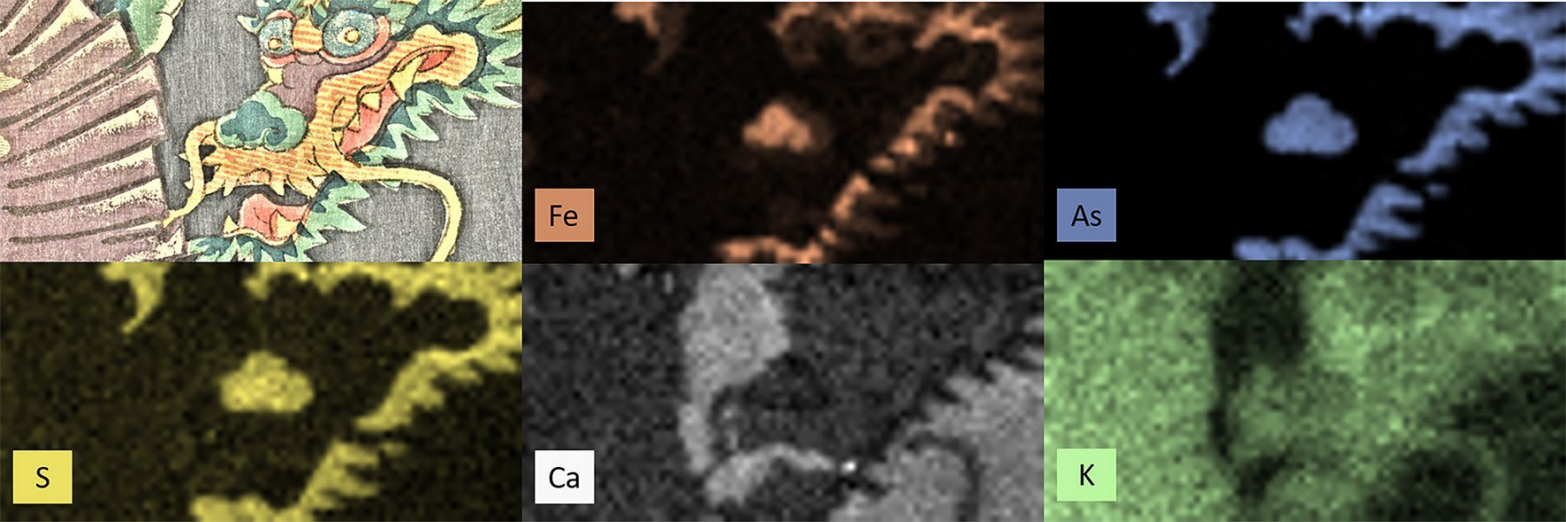
MA-XRF elemental maps for print A, showing the distribution of iron (Fe, brown), arsenic (As, blue), sulfur (S, yellow), which were identified in the green areas of the print. Calcium (Ca, white) was identified in the dark blue background. Potassium (K, green) was found in all the areas where calcium was not observed. The elemental maps were acquired using the Elio software and elaborated using PyMCA software suite (version 5.5.0).

**Figure 4:**
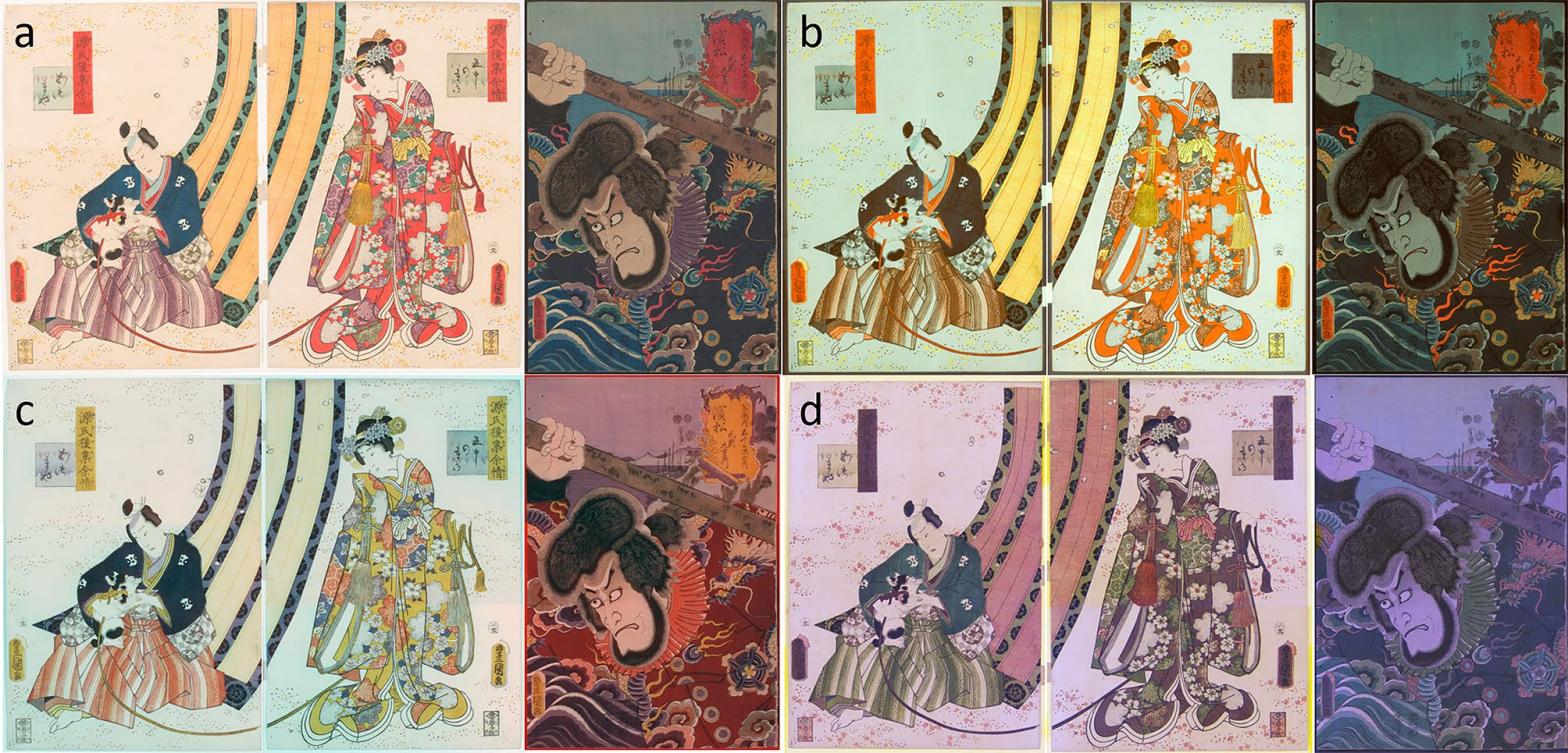
MSI of prints A and B (**a**) VIS, (**b**) UVL, (**c**) IRRFC and (**d**) UVRFC. All images were captured using the Camera Pro 2 software and false color images were created using Adobe Photoshop version 20.0.9.

All MA-XRF elemental maps obtained for both prints A and B are given in Figs. 2, 3 and Fig. S3, and the MSI images in Fig. 4.

MA-XRF enabled some red, green and blue inorganic pigments to be tentatively identified. The presence of iron (Fe) in the red areas of print B (Fig. 2-Fe) indicated that an iron-based pigment, most likely a red ochre, was used. Nonetheless, Fe was also found in the blue and green areas of both prints (Fig. 2-Fe and Fig. 3-Fe). In the blue areas, the use of Prussian blue (Fe[Fe^3+^Fe^2+^(CN)_6_]_3_) was hypothesized. Similarly, this pigment was suspected in the green areas as well, especially considering that As and S were also present in all these areas (Fig. 2-As, Fig. 3-As and Fig. 3-S), thus indicative of a mixture with a yellow arsenic sulfide pigment. This mixture is in agreement with the findings of previous studies on Japanese woodblock prints^4,12,14,15^. However, the presence of Fe in green areas could also be in principle associated with green earth (K[(Al,Fe^3+^),(Fe^2+^,Mg](AlSi_3_,Si_4_)O_10_(OH)_2_). This pigment is not reported in any study of Japanese woodblock prints but its presence cannot be excluded on the sole basis of MA-XRF data.

Lead (Pb) was found in the blue area of the man’s kimono (Fig. 2-Pb). This was an unexpected finding, as Pb is usually associated with lead white (2PbCO_3_·Pb(OH)_2_) or red lead (Pb_3_O_4_), and therefore not associated with a dark blue pigment. In this particular case, lead white was most likely used with Prussian blue, either in mixture to tone down the dark blue hue of the Prussian blue, or as a first layer to give a fully reflective base to the blue pigment and therefore increase the brilliance of the upper blue pigment layer.

Copper (Cu) and potassium (K) were also identified in print B, especially in the green cartouche of the right-hand side print (Fig. 2-Cu and Fig. 2-K). This indicated the use of a potassium-rich copper-based green. By contrast, the other cartouche, in the left-hand side print, did not contain these elements. Both cartouches, however, contained a mixture of Fe-based blue (most likely Prussian blue, Fig. 2-Fe) and As-based yellow (Fig. 2-As), especially in the darker green highlights in the bottom half of the cartouches. Although the Cu-rich green pigment might be associated with a later conservation treatment, the Cu/K difference between the two cartouches, along with the variation in As and Fe intensities in the green parts of the curtains and in the red cartouches in print B (Fig. 2-Fe and Fig. 2-As), might indicate that the two impressions were realized in different workshops. In fact, the practice of sending composed prints to more than one studio is known during the Meiji period, with the aim to avoid non-authorized full copies to be created. This case might represent an early example of such practice.

Calcium (Ca) was present in the dark blue areas of print A (Fig. 3-Ca) and could be associated with bone black, although P was not identified, and the pigment was not confirmed by Raman analysis. For print B, Ca is most likely associated with the mounting board, as shown in Fig. S3. Potassium (K) was identified throughout both prints, regardless of the presence of color (Fig. 2-K, Fig. 3-K), except in the dark blue areas of print A. K is likely to be associated with paper-sizing, especially considering its presence in the uncolored areas of print B (Fig. 2-K) and the lack of intensity variation among the colored and uncolored areas in Print A (Fig. 3-K). In the hypothesis of the organic colorants being precipitated as lakes on a K-containing substrate, such variations should have been detected.

For both prints, all other colors, including bright yellow, lighter shades of red and various shades of purple, did not yield any elemental signal, which suggested the use of organic colorants. As MSI is well suited for the investigation of some organic colorants^14,19,20^, the images obtained provided further information.

MSI is based on the principle that most materials have a specific response to different wavelengths in terms of reflection and absorption, allowing us to distinguish some pigments and colorants. Previous studies have reported the color combinations exhibited by certain organic colorants in the various MSI images obtainable, in particular VIS, UVL, IRRFC and UVRFC^14,19,20^. Our results were interpreted by comparison with these data and making sure that the images were acquired and processed in standardized ways^37^.

All red areas of the prints exhibited an intense orange UV-induced visible luminescence (Fig. 4b). The same areas were shown as yellow in IRRFC images (Fig. 4c) and as dark purple in UVRFC images (Fig. 4d). This color combination is characteristic of the plant-based organic red colorant derived from safflower (*Carthamus tinctorius*), whose main chromophore is carthamin. Although no other red colorant exhibits such intense UV-induced visible luminescence^20^, further molecular confirmation is generally needed to support MSI interpretation. In print B, when the distribution of safflower red (Fig. 4b) was compared with the distribution of Fe obtained by MA-XRF (Fig. 2-Fe), it was noticed that the organic colorant was found along with the Fe-containing red pigment, most likely red ochre, in most red areas, with the exception of the borders of the man kimono (around the foot and around the neck) and the hair pins of the woman. These areas did not contain any Fe and appear pink in the VIS image (Fig. 4a), suggesting the use of sole safflower red to obtain this color shade. In print A, such considerations were more difficult to obtain, because MA-XRF was only performed on the dragon-head detail, in which safflower red was clearly present on the tongue and around the eyes. Nevertheless, single spot XRF analyses on this print (not shown) revealed the presence of Fe in the cartouche, therefore suggesting the use of a mixture of Fe-containing red pigment, likely red ochre, and safflower red in this area. From a methodological point of view, it was also important to notice that safflower red retained its UV light-induced luminescence properties even if mixed with an inorganic Fe-containing red pigment. The intense orange color of the UV-induced luminescence slightly changed—it was slightly darker—compared to safflower red alone, but it remained unmistakable for the identification of this colorant. This also opens possibilities for further investigations about how the colors are mixed (over-printed vs. mixed) and how different mixing methods may affect the photo-physical properties of the colorants.

The yellow color found in the squares in the background, curtains, kimono and yellow cartouche of print B, as well as in the dragon’s head and yellow details of the kimono in print A, exhibited an intense UV-induced visible luminescence in the yellow range (Fig. 4b). These areas appeared creamy whitish yellow in IRRFC images (Fig. 4c) and red/brown in UVRFC images (Fig. 4d). This color sequence is characteristic of some organic yellows, such as turmeric (*Curcuma longa*, the main chromophores are curcumin, methoxycurcumin and demethoxycurcumin) and amur-cork tree (*Phellodendron amurense*, the main chromophore is berberine). It is however difficult to distinguish them based solely on MSI.

The dark blue shade in the kimono of print A exhibited a different behavior from the other blue shades in this print, as observed in the IRRFC image (Fig. 4c). The false red color of this area was indicative of the use of indigo.

The purple color found in both prints appeared brownish in UVL images (Fig. 4b), orange in IRRFC images (Fig. 4c) and green in UVRFC images (Fig. 4d). The false colors were in partial agreement with the possible presence of gromwell (*Lithospermum erythrorhizon*, the main chromophore is shikonin), but this colorant is known to completely absorb the UV radiation. As a consequence, the color combination in the various imaging modes did not precisely correspond to any specific colors previously described and published. The lack of bromine (Br) in MA-XRF enabled the possibility of Tyrian purple to be discarded, although it would have been very unlikely to find such precious colorant in Japanese prints and there is no record of its use in such art form. In the hypothesis of a mixture of red and blue organic colorants, the presence of indigo was difficult to assess, as the orange hue showed in the IRRFC image was slightly too light to justify the presence of indigo. Dayflower would also represent a possible option for the blue component, but it is supposed to exhibit a light green UV-induced luminescence and to appear as pink in IRRFC images. The use of safflower red as the red component was also not justified by the lack of the characteristic intense orange UV-induced luminescence.

While providing valuable information about the palette used in the woodblock prints under investigation (Table 1), MA-XRF and MSI did not allow us to conclude on the nature of most organic colorants, especially yellow and purple, hence further spectroscopic single spot or minimally invasive analyses were required.

**Table 1:** Summary of the multispectral imaging and MA-XRF results for prints A and B.

Print	MSI colors				MA-XRF	Possible identification
	VIS	UVL	IRRFC	UVRFC		
15.07h Kunisada 1852 (Print A)	Blue	Greyish blue	Purple	Blue	N/A	Prussian blue
	Dark blue	Black	Red	Blue	Ca	Indigo or dayflower blue Carbon black
	Red	Orange	Yellow	Purplish brown	N/A	Organic red
	Yellow	Yellow	White	Reddish brown	N/A	Organic yellow
	Green	Greenish blue	Purple	Olive green	Fe As S	Prussian blue + arsenic sulfide
	Purple	Brown	Orange	Green	N/A	Organic purple or mixture of organic blue and red
JP3683a,b Kunisada 1858 (Print B)	Blue	Dark grey	Dark blue	Greenish blue	Fe Pb	Prussian blue (MA-XRF/MSI) Lead white (MA-XRF)
	Red	Orange	Yellow	Purplish brown	Fe	Red ochre (MA-XRF) Safflower (MSI)
	Yellow	Yellow	White (squares) Yellow (fabric)	Reddish brown	N/A	Turmeric or amur-cork tree (MSI)
	Green	Greenish blue	Greyish blue	Olive green	Fe As	Prussian blue + arsenic sulfide (MA-XRF/MSI)
	Purple	Brown	Orange	Green	N/A	Organic purple or mixture of organic blue and red

### Spectroscopic single spot analyses: Raman and FORS

The results obtained by single spot FORS and Raman analyses on the various colored areas of the prints are summarized in Table 2.

**Table 2:** Summary of the FORS and Raman results for prints A and B.

Print	Color	FORS		Raman shift (cm^−1^)	Identification (tentative are marked with an asterisk *)
		Inflection point (nm)	Maximum absorption (nm)		
15.07h Kunisada 1852 (Print A)	Blue	Featureless—slope (650–950)		2095, 2155	Prussian blue
	Red	580	525, 675, 855	227, 294, 412, 614	Safflower red Red ochre
	Yellow	–	–	–	Organic yellow
	Green	470	–	151, 217, 233, 334, 472, 493, 2092, 2155	Prussian blue + amorphous arsenic sulfide
	Purple	–	535, 590, 640	-	Dayflower blue* + safflower red*
JP3683a,b Kunisada 1858 (Print B)	Blue	Featureless—slope (650–950)		2095, 2155	Prussian blue
	Dark blue	715	660	1572, 1583	Indigo
	Red	–	525	–	Safflower red
	Yellow	–	–	–	Organic yellow
	Green	470	–	233, 334, 472, 493, 2092, 2155	Prussian blue + amorphous arsenic sulfide
	Purple (dark shade)	–	535, 590, 640	–	Dayflower blue* + safflower red*
	Purple (light shade)	–	525, 640	–	Unknown

**Figure 5:**
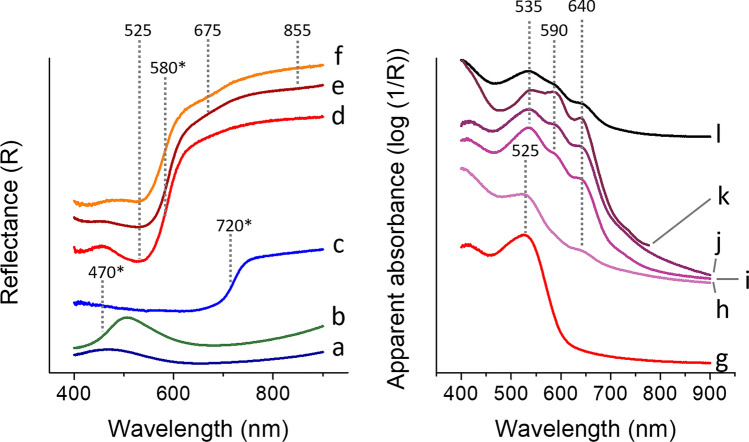
Reflectance (left) and apparent absorption (right) spectra obtained by FORS for representative colors found in prints A, B and C: (a) blue—Prussian blue; (b) green—mixture of Prussian blue and unidentified yellow; (c) dark blue—indigo; (d, g) light red—safflower red; (e, f) dark red—safflower red and red ochre; (h) light purple—unidentified; (i, j, k) medium and dark purple—possible mixture of safflower red and dayflower blue; (l) linear combination of the reflectance spectra for safflower red and dayflower blue. (k) is the FORS spectrum obtained for print C. Asterisks (*) indicate values of the inflection points while other values indicate maximum absorption.

**Figure 6:**
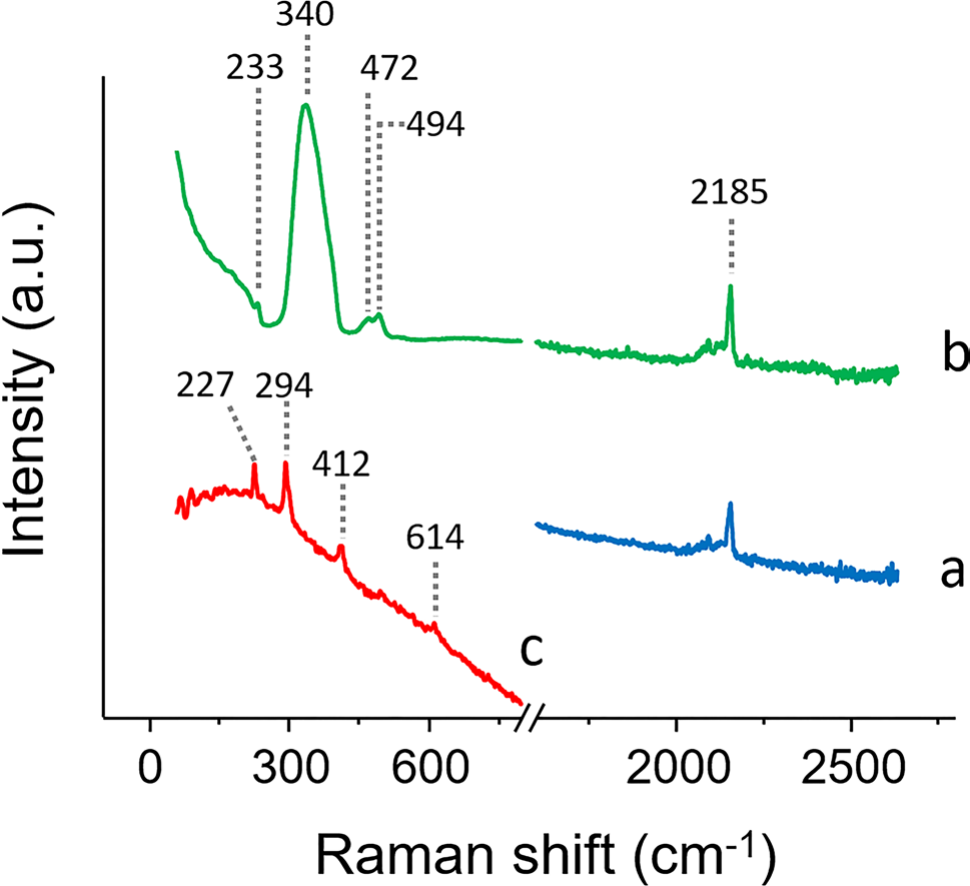
Raman spectra obtained for representative colors found in prints A and B: (a) blue—Prussian blue; (b) green—Prussian blue and amorphous arsenic sulfide; (c) red—red ochre.

Raman and FORS spot analyses on the prints confirmed the use of Prussian blue in the blue areas of both prints (Figs. 5a and 6a), as well as in the green areas, where it was in mixture with yellow (Figs. 5b and 6b), identified as artificial arsenic sulfide by Raman spectroscopy and not natural orpiment (Fig. 6b)^46^. In print A, FORS also enabled indigo to be confirmed in the darkest shade of blue, as the spectra were characterized by the typical inflexion point at *ca.* 720 nm and the lack of spectral features in the 550–675 nm range (Fig. 5c). The maximum absorption at 525 nm observed in both reflectance and apparent absorbance FORS spectra of the red areas of both prints (Fig. 5d,g) enabled the presence of safflower red to be confirmed. Both techniques also allowed us to confirm the presence of red ochre along with safflower red in the red areas of print B (Figs. 5e,f and 6c), based on the combined presence of the 525 nm maximum absorption for safflower red and the 675 and 855 nm maximum absorptions and 580 nm inflection point, all three characteristic for iron oxide pigments.

The purple areas did not yield any suitable Raman spectra. This indicated that indigo was not likely to be used. FORS of the various purple shades yielded slightly different spectra (Fig. 5h–j). The darkest areas presented three absorption maxima at 535, 590 and 640 nm (Fig. 5i,j), whereas the lighter shades presented only two absorption maxima at 525, and 640 nm (Fig. 5h). The three absorption maxima observed for the darker shades of purple appeared to be common to several purple colorants or mixtures, such as Tyrian purple, orchil, Tyrian purple/anthraquinone or dayflower/safflower mixtures^17,54,55^. While the use of Tyrian purple was very unlikely due to the lack of bromine in XRF maps, the identification of the purple colorant remained uncertain. The use of the purple yielding the three absorption maxima at 535, 590 and 640 nm was also found on a print (print C) dated 1786 (Fig. 5k). This indicated that the colorant or mixture is at least consistent throughout time in Edo-period Japan. When compared to a linear combination of the reflectance spectra for safflower red and dayflower blue (Fig. 5l), a close match was obtained. This most likely indicated that the color used in the darkest shades of purple was a mixture of dayflower blue and safflower red. Nonetheless, the absorption maximum at 590 nm was absent in the medium and light shades of purple in print B, and the 535 nm apparent absorption band was shifted down to 525 nm in the lighter shade of purple. These variations made the identification of the dayflower blue/safflower red mixture in these lighter areas of the prints (or in other prints presenting only light shades of purple) not straightforward. Therefore, micro-invasive analyses were considered.

### Micro-invasive analyses: SERS and HPLC-ESI-Q-ToF

**Figure 7:**
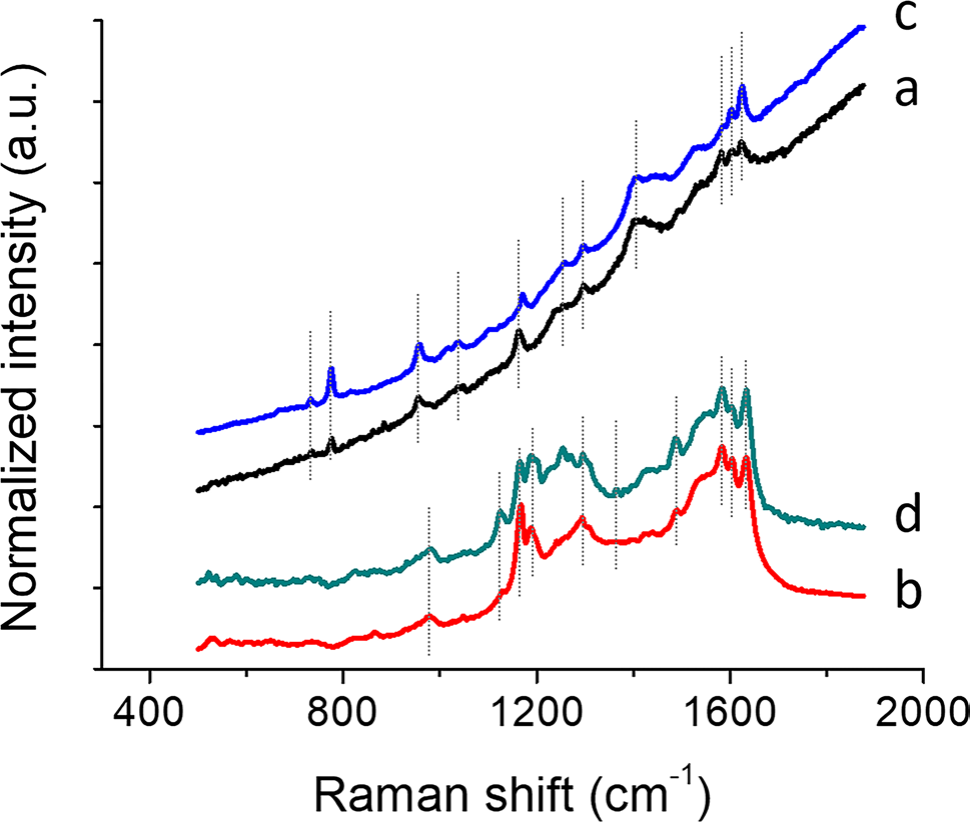
SERS spectra for (a) red (sample 03 and 06) and (b) yellow (samples 01 and 05) areas of prints A and B presented along with (c) safflower red and (d) yellow turmeric reference spectra.

Micro-invasive analyses were mainly used to confirm or identify the organic colorants used in the prints. Due to its previous use for colorant identification in Japanese artifacts^11,14,30^, SERS was initially applied. SERS allowed for a positive identification of safflower red and turmeric, respectively in the red and yellow areas of the prints (Fig. 7). The purple colorant (or blue and red mixture, sample 02) was more challenging and did not yield any positive identification using SERS. Interestingly, a clear modification of the purple color was observed upon acidic hydrolysis. The fiber turned from deep purple to bright red. SERS analyses on hydrolyzed and non-hydrolyzed samples did not yield any spectroscopic signals, neither for a pure purple, as it appears under microscopic observation, nor for mixtures of red and blue colorants, as it could be suggested by the color shift following the sample hydrolysis. This was somehow peculiar, as most red colorant are usually quite easy to identify using SERS^28^. Therefore, HPLC-ESI-Q-ToF was considered for its power to provide information at a molecular level.

The chromatographic profiles for samples 04, 07 and 08, corresponding to two dark and one light shades of purples, were acquired in both negative and positive ionization modes. They showed the presence of several molecules and the chemical composition and chromatographic profiles were very similar for all samples. This accounted for the very high sensitivity of the technique and therefore the successful detection of such molecules starting from the small amount of sample used. It is worth mentioning that fibers from a print may have a relatively high concentration of pigment/colorant deposited on the surface as a result of the printing process, thus enhancing the possibility of molecular detection from a very small sample. This situation is not directly comparable to textile fibers, in which the dyes are applied differently (direct, mordant or vat) and interact with the fibers in complex ways, generally resulting in lower concentrations of the dye molecules.

Among the detected molecules, carthamin (C_43_H_42_O_22_, [M−H]^−^ = *m/z* 909.2095) was only detected in negative ionization mode and was identified by comparing the results with those present in an in-house molecular database^34^. This molecule is the molecular marker for safflower red (*Carthamus tinctorius*)^56^. When safflower is present in a sample, carthamin is generally accompanied by at least two other molecules, which have been identified as carthamin degradation products by retro-aldol reaction^34,57^. It was interesting to notice that these molecules were not present in these samples, suggesting a very good preservation state of the red color in these prints, especially considering the poor stability of carthamin within a wide range of conditions, including not only light exposure, but also humidity, temperature and ozone^58^.

**Figure 8:**
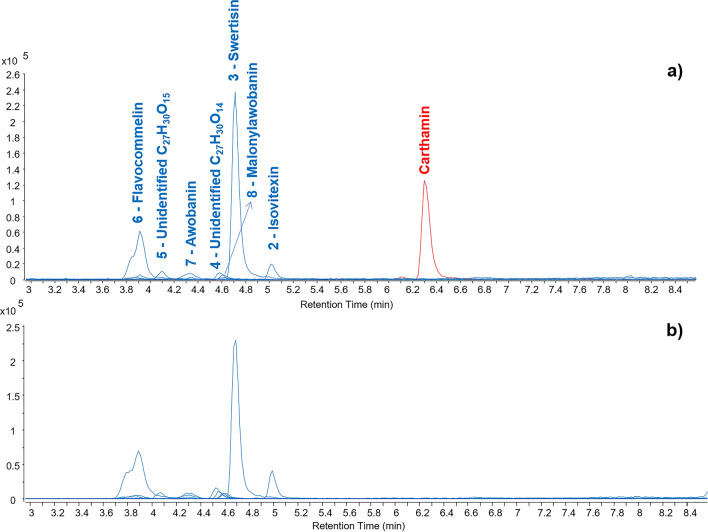
Extract ion chromatograms of the molecules detected by HPLC-ESI-Q-ToF analysis (negative mode) of (**a**) sample 04 and (**b**) a reference of dayflower blue.

The other molecules were detected in both negative and positive ionization modes. The distribution of these molecules perfectly matched with the results obtained for the reference sample of paper dyed with dayflower blue (*Commelina communis*), as shown in Fig. 8 and Fig. S4 (Supplementary Information).

Although the molecular composition of dayflower blue is known^59–61^, this is the first account of its identification by HPLC-ESI–Q-ToF from a work of art and the first account of the mass spectrometric details of all its molecular components in such context (Table 3). The blue color of dayflower is primarily given by commelinin, which is a metalloanthocyanin, i.e. a self-assembled, supramolecular metal complex pigment composed of an anthocyanin (malonylawobanin, i.e. delphinidin-3-O-[6-O-(*p*-coumaroyl)-β-d-glucopyranoside]-5-O-[6-O-malonyl-β-d-glucopyranoside]), a flavone (flavocommelin, i.e. 7-O-methylapigenin-6-C-glucopyranoside-4′-O-β-d-glucopyranoside), and a metal ion (Mg^2+^) in the stoichiometric ratios 6:6:2^59^. Malonylawobanin and flavocommelin were both identified in the samples analyzed. In addition, awobanin (delphinidin-3-O-[6-O-(*p*-coumaroyl)-β-d-glucopyranoside]-5-O-β-d-glucopyranoside], swertisin (7-O-methylapigenin-6-C-glucopyranoside), vitexin (apigenin-8-C-glucopyranoside) and isovitexin (apigenin-6-C-glucopyranoside) were also detected in both the reference sample and the two samples from the prints. While awobanin directly relates to the structure of malonylawobanin, the other molecules, such as swertisin, vitexin and isovitexin are apigenin-based flavonoids, but they are also naturally present in the extracts of *C. communis*^61^. The results also matched with those available in the literature about MS fragmentation of some of these molecules^62,63^. Two additional unidentified molecules were present in all samples and the molecular structure was closely related to flavocommelin according to the MS fragmentations obtained. The mass spectrometric results for these molecules, including fragment ions obtained in positive and negative MS/MS experiments are reported in Table 3. The MS/MS spectra and molecular structures are reported in Supplementary Information (Figs. S5–S10).

**Table 3:** Mass spectrometric details of the main components of dayflower blue detected by HPLC-ESI-Q-ToF.

No	Name	Formula	MW	MS neg (m/z)	MS/MS neg (m/z)^a^	MS pos (m/z)	MS/MS pos (m/z)^a^
1	Vitexin	C_21_H_20_O_10_	432.106	[M−H]^−^ = 431.098		[M+H]^+^ = 433.113	
2	Isovitexin	C_21_H_20_O_10_	432.106	[M−H]^−^ = 431.098		[M+H]^+^ = 433.113	
3	Swertisin	C_22_H_22_O_10_	446.121	[M−H]^−^ = 445.114	325, 297, 282, 269,	[M+H]^+^ = 447.129	429, 411, 393, 375, 351, 327, 297
4	Unidentified	C_27_H_30_O_14_	578.164	[M−H]^−^ = 577.156	487, 457, 413, 341, 311, 293	[M+H]^+^ = 579.171	433, 415, 397, 379, 367, 337, 313
5	Unidentified	C_27_H_30_O_15_	594.159	[M−H]^−^ = 593.151	503, 473, 413, 383, 353, 311, 297, 282	[M+H]^+^ = 595.168	577, 559, 541, 539, 511, 499, 475, 457, 427, 397, 379, 337, 313, 283
6	Flavocommelin	C_28_H_32_O_15_	608.174	[M−H]^−^ = 607.167 [M+Cl]^−^ = 643.144 [M+COOH]^−^ = 653.172	487, 455, 325, 309, 293, 281	[M+H]^+^ = 609.181	591, 573, 555, 543, 513, 489, 459, 429, 411, 393, 381, 351, 327, 297
7	Awobanin	C_36_H_37_O_19_^+^	773.193	[M−H]^−^ = 771.178 [M+H_2_O−H]^−^ = 789.188 [M+Cl]^−^ = 807.155 [M+COOH]^−^ = 817.183	Not obtained	[M+H]^+^ = 773.193	611, 465, 303
8	Malonylawobanin	C_39_H_39_O_22_^+^	859.193	[M−H]^−^ = 857.178 [M+H_2_O–H]^−^ = 875.189	Not obtained	[M+H]^+^ = 859.193	611, 551, 303

^a^Nominal masses of the fragment ions are reported in the table. Refer to Supplementary Information for accurate masses.

It is also important to underline that the molecules showed very different ionization yields in positive and negative mode. Anthocyanins are known to be difficult candidates for detection in negative ionization mode, due to their tendency to form water adducts and other types of adducts, thus complicating the MS identification^64^, whereas they ionize more easily in positive mode. As a result, awobanin and malonylawobanin showed a complicated mass spectrum in negative mode and a higher relative abundance in positive mode. A reliable MS/MS spectrum was only obtained in positive mode for these molecules. A significant difference between ionization modes was also observed for flavocommelin. Similarly to the anthocyanins, a complicate mass spectrum was obtained in negative mode and a much higher relative abundance was obtained in positive mode, but a reliable MS/MS spectrum was obtained in both ionization modes.

These results suggested that the positive ionization mode would be more appropriate for the detection of dayflower molecular markers by HPLC-ESI-Q-ToF. Nevertheless, in this specific case, carthamin would not be detected in positive mode. Therefore, both ionization modes should always be adopted in similar situations.

Despite inconclusive MSI observations, clear variations in FORS spectra and lack of signal in Raman and SERS spectra for the purple areas, HPLC-ESI–Q-ToF unambiguously identified both shades of purple found in both Kunisada prints (print A and B) as a mixture of safflower red and dayflower blue. The technique also allowed us to conclude that the prints were in an extremely well-preserved state, as both colorants used for the purple were not degraded. This information is of remarkable importance in order to understand the degree of light-induced degradation of prints for acquisition, conservation or exhibition purposes.

In the case of the purple colorant of the prints, we noted an interference effect of dayflower on the electronic and vibrational features of carthamin, which affected MSI and SERS detection. In both cases, and contrary to our experience with the pure safflower dye, we were not able to observe the typical features of carthamin. The investigation of the photo-physical and spectroscopic properties of organic colorants when applied in mixtures is a topic that requires additional research, which was not undertaken here, as outside of the scope of this specific study, but deserves attention in the future.

On an art historical note, the finding of safflower red and dayflower blue used in a mixture to obtain purple in late Edo period prints is also quite significant. Indeed, the late Edo period saw the introduction of new pigments in the Japanese prints’ palette, including Prussian blue and amorphous arsenic sulfide. In the 1850s, the occurrence of dayflower blue or even indigo is very sparse. Dayflower blue was and is well-known for its instability, and most blue shades were obtained using Prussian blue. However, the mixture of safflower red and dayflower blue was found in prints created earlier in the Edo period^2^, including the third print under consideration here (print C), dated around 1786, as suggested by the similarity of its FORS spectrum with the one for the dark shades of the two other prints (Fig. 5k). Furthermore, the combined use of dayflower blue and safflower red to yield the purple color found in the prints is consistent with Yamato’s survey of Edo period prints using noninvasive techniques, highlighting the use of traditional colorants up to the end of the Edo period^65^. This shows how Japanese artists and print manufactures remained attached to traditional materials when it comes to certain colors.

## Conclusions

This work has demonstrated that the characterization of mineral pigments and organic colorants in Japanese woodblock prints is a challenging process that requires a multi-analytical approach. MA-XRF, applied for the first time to the study of coloring materials in Japanese woodblock prints, clearly showed its great potential for the identification and visualization of the spatial distribution of inorganic pigments in the prints. MSI confirmed its complementarity in terms of identification and visualization of the spatial distribution of selected organic colorants, in particular safflower red and indigo. Therefore, the combination of these two techniques appear as the best initial approach to be applied in an analytical protocol tailored for Japanese woodblock prints. Nonetheless, both techniques clearly showed shortcomings regarding the characterization of organic colorants, pure or in mixtures, especially in the purple areas.

The application of single point non-invasive techniques, such as Raman and FORS, also confirmed the potential of these methods and, in some cases, their complementarity for inorganic pigments and organic colorants respectively. While both techniques mostly confirmed the finding from imaging techniques, FORS also suggested a mixture of dayflower blue and safflower red used in the dark purple areas. Nonetheless, clear variations in FORS spectra between the various hues and inconclusive MSI observations made the identification based on FORS uncertain. Furthermore, single point analyses did not provide much more information than MSI on the nature of most organic colorants.

Minimally invasive techniques, such as SERS and HPLC–MS/MS were applied on selected micro-samples. While SERS positively identified the turmeric, it did not yield any positive identification of the purple mixture, hence the introduction of HPLC–MS/MS in the analytical protocol, presented here for the first time in the study of colorants in Japanese prints. The technique allowed us to positively identify the components of the purple colorant as a mixture of safflower red and dayflower blue in both dark and light shades. In addition to the identification of the colorants, HPLC–MS/MS also indicated that the prints were in an extremely well-preserved state, as both colorants used for the purple were not degraded. This information is of remarkable importance to understand the degree of light-induced degradation of prints for acquisition, conservation or exhibition purposes. The positive results clearly showed that HPLC–MS/MS can be included into analytical protocols applied to works on paper, when a few fibers yielding a significant concentration of colorant can be peeled off.

Finally, the palette used in the various prints was in agreement with what is expected for late Edo period prints, except for the dayflower blue and safflower red mixture found in the purple colorant. Dayflower blue is often considered a colorant used early in the print production time, but, as demonstrated in this study, the purple color composition remained the same over time in Japan, while other colors, including blues and yellows, evolved. Interestingly, dayflower blue, even though theoretically replaced by the more flexible Prussian blue in late Edo prints, actually remained in use alongside Prussian blue and/or indigo. This proves that, despite the apparent evolution of materials and techniques happening in Japan in the late Edo period, some colorants and techniques remained traditional, highlighting the ambiguity of Japan during this great period of change, when the country was leaning toward modernization and the West, while keeping its traditional grounds.

## Supplementary information

Supplementary Information.

## Data Availability

All data generated or analyzed during this study are included in this published article and in its Supplementary Information file.
