# Supplementary material for: Integrating liquid chromatography mass spectrometry into an analytical protocol for the identification of organic colorants in Japanese woodblock prints
Source: Sci Rep. 2020 Dec 1;10:20921. doi: 10.1038/s41598-020-77959-2 (PMC7708457; doi:10.1038/s41598-020-77959-2)
Supplement: Supplementary file 1 — Supplementary Information. [file 41598_2020_77959_MOESM1_ESM.pdf]

Supplementary Information

Traditional purple in times of change – integration of high-pressure liquid chromatography mass spectrometry into an analytical protocol for the identification of organic colorants in Japanese woodblock prints

Marc Vermeulen[1,a*], Diego Tamburini[2,b], Emilie M. K. Muller[3], Silvia A. Centeno[1], Elena Basso[1], Marco Leona[1]

[1] Department of Scientific Research, The Metropolitan Museum of Art, 1000 Fifth Avenue, New York, NY 10028, USA
[2] Department of Scientific Research, The British Museum, Great Russell Street, London WC1B 3DG, UK
[3] Department of Paper Conservation, The Metropolitan Museum of Art, 1000 Fifth Avenue, New York, NY 10028, USA

[a] Current affiliation: Northwestern University – Art Institute of Chicago Center for Scientific Studies in the Arts (NU-ACCESS), 2145 Sheridan Road, Tech K111, Evanston, IL 60208, USA
[b] Current affiliation: Department of Conservation and Scientific Research, Freer Gallery of Art and Arthur M Sackler Gallery, Smithsonian Institution, 1050 Independence Ave SW, Washington DC, 20560, USA

* Corresponding author: Dr Marc Vermeulen; email: marc.vermeulen@northwestern.edu

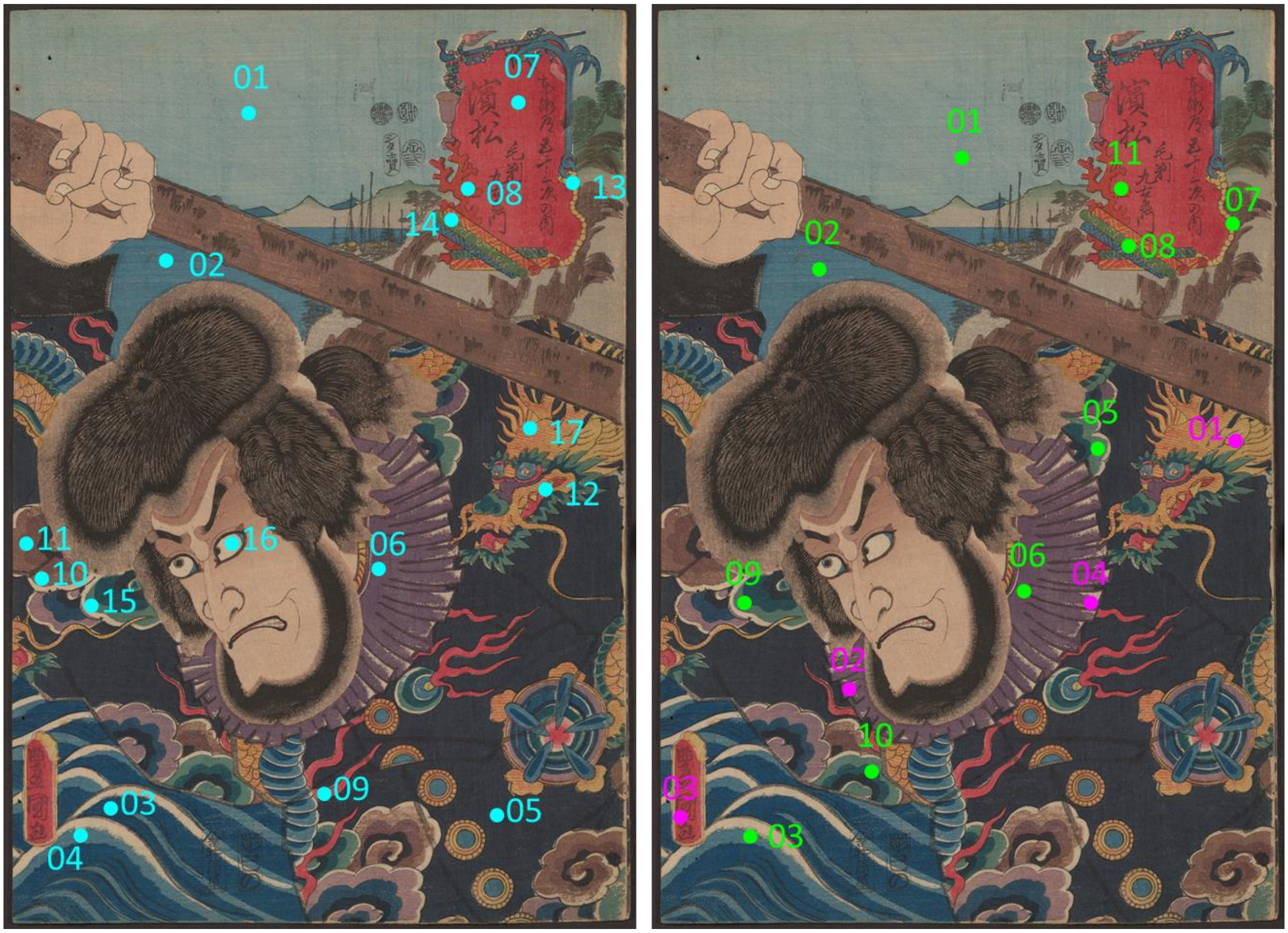

Figure S1. Analyses spot locations of print A for FORS (cyan), Raman (lime) and sampling locations for SERS and HPLC-MS/MS (magenta). Collection of Henry D. Smith II. Image: The Metropolitan Museum of Art.

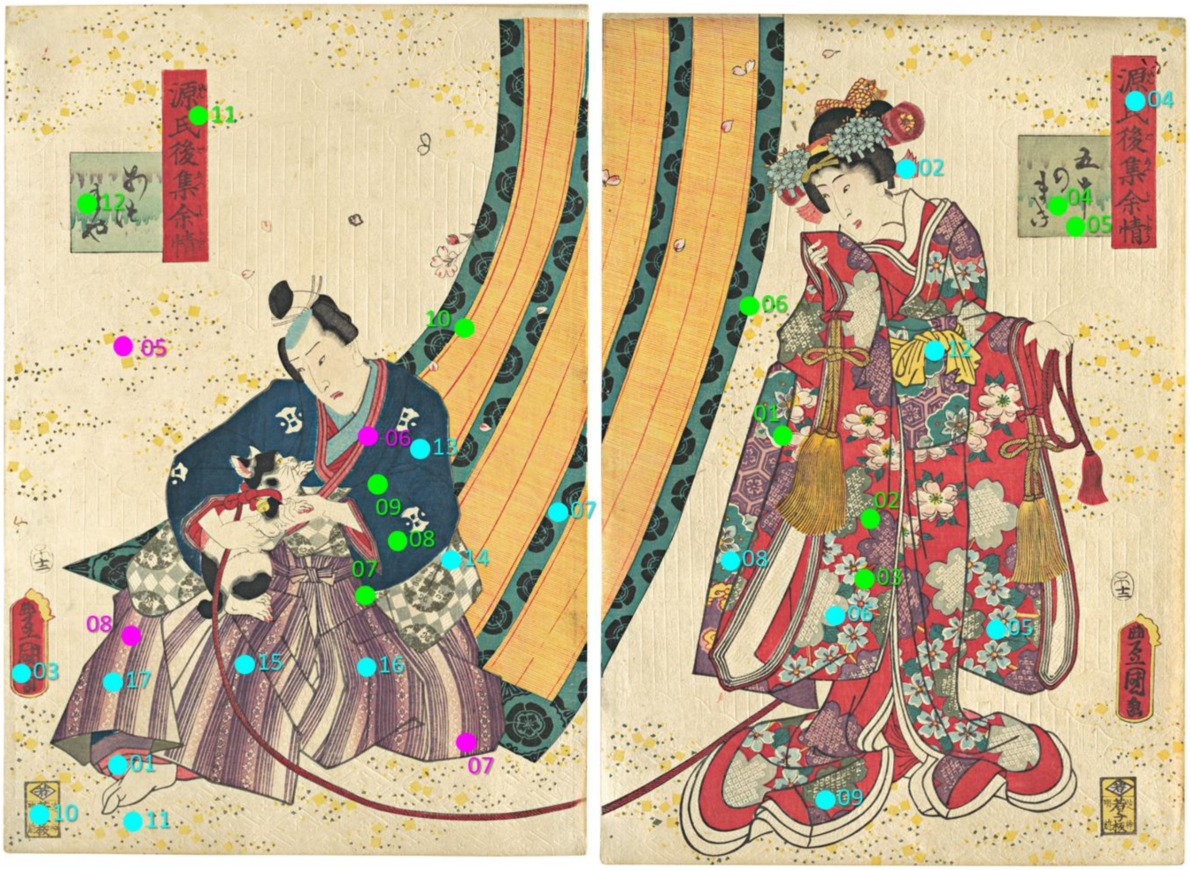

Figure S2. Analyses spot locations of print B for FORS (cyan), Raman (lime) and sampling locations for SERS and HPLC-MS/MS (magenta). The Metropolitan Museum of Art, Gift of Lincoln Kirstein, 1985.

Table S1. List of samples taken from prints A and B for SERS and HLPC-MS/MS analyses.

Sample	Print	Color	Technique used
01	A	Yellow	SERS
02	A	Dark purple	SERS
03	A	Red	SERS
04	A	Purple	HPLC-ESI–Q-ToF
05	B	Yellow	SERS
06	B	Red	SERS
07	B	Dark purple	SERS/HPLC-ESI–Q-ToF
08	B	Light purple	HPLC-ESI–Q-ToF

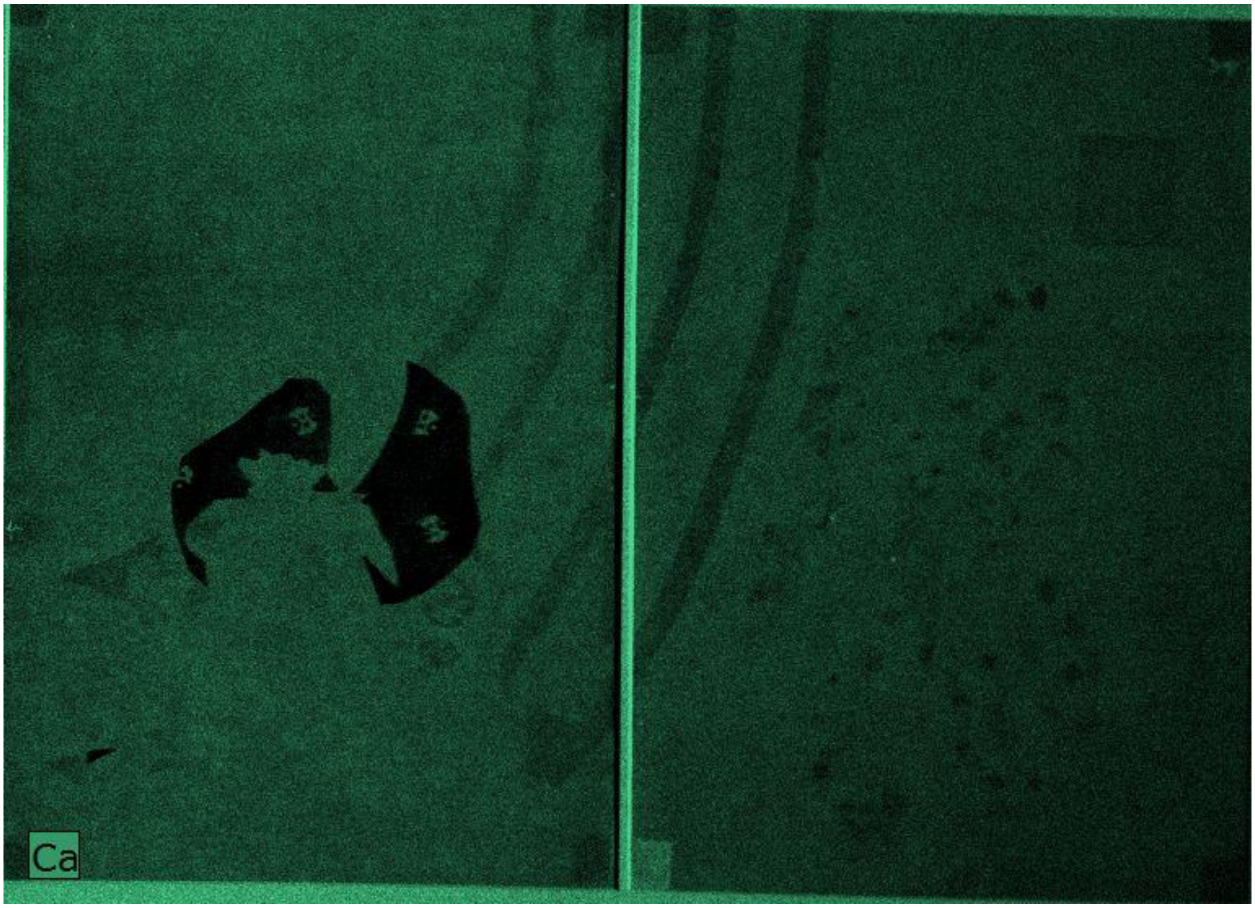

Figure S3. MA-XRF elemental maps for print B, showing the distribution of calcium (Ca). The higher intensity for Ca observed around and in between the prints indicates that Ca is most likely found in the mounting material. Its presence throughout the prints except in areas where high-Z elements are found (arsenic, lead, copper), may indicate the presence of Ca in the paper itself. The elemental map was created using the Bruker M6 software package.

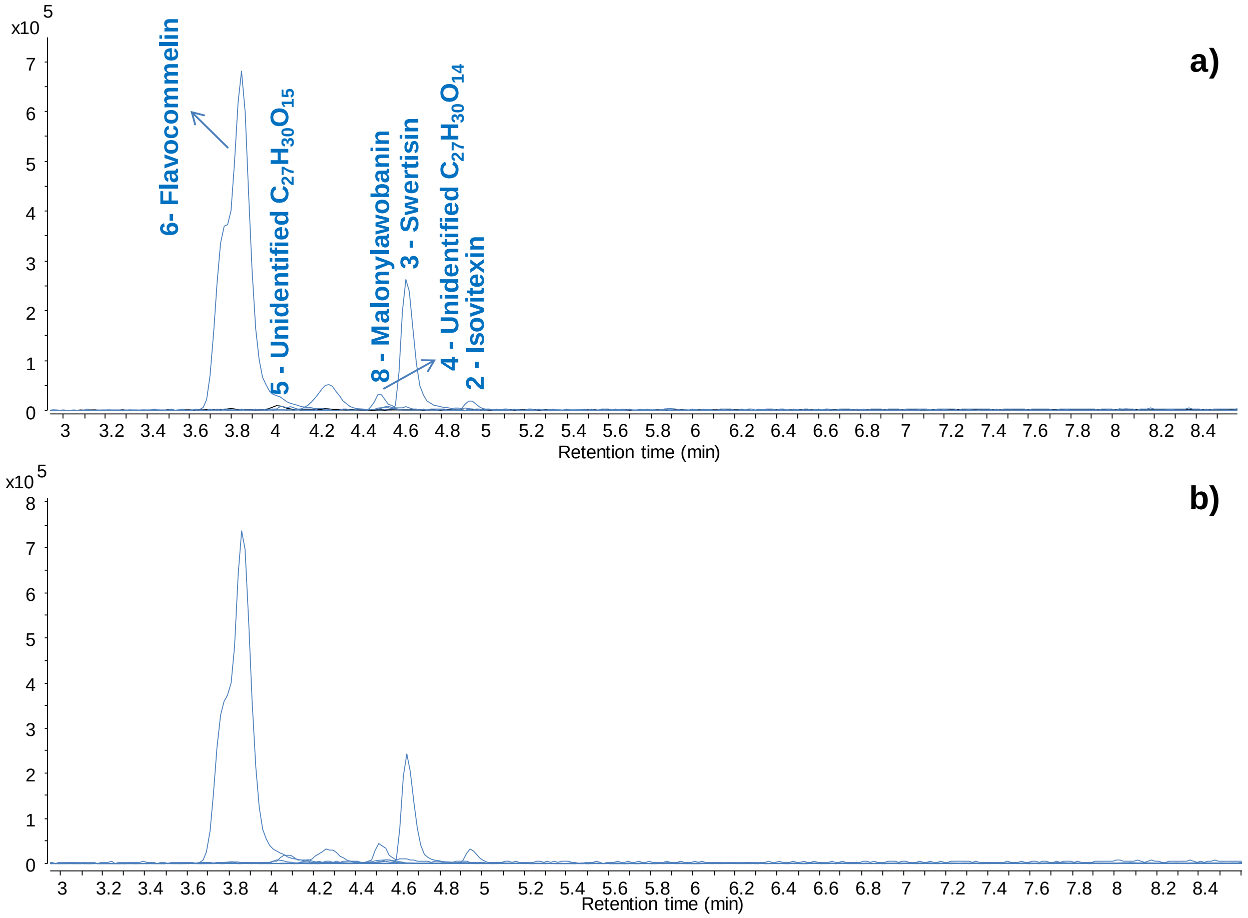

Figure S4. Extract ion chromatograms of the molecules detected by HPLC-ESI-Q-ToF analysis (positive mode) of **a)** sample 04 and **b)** a reference of dayflower blue.

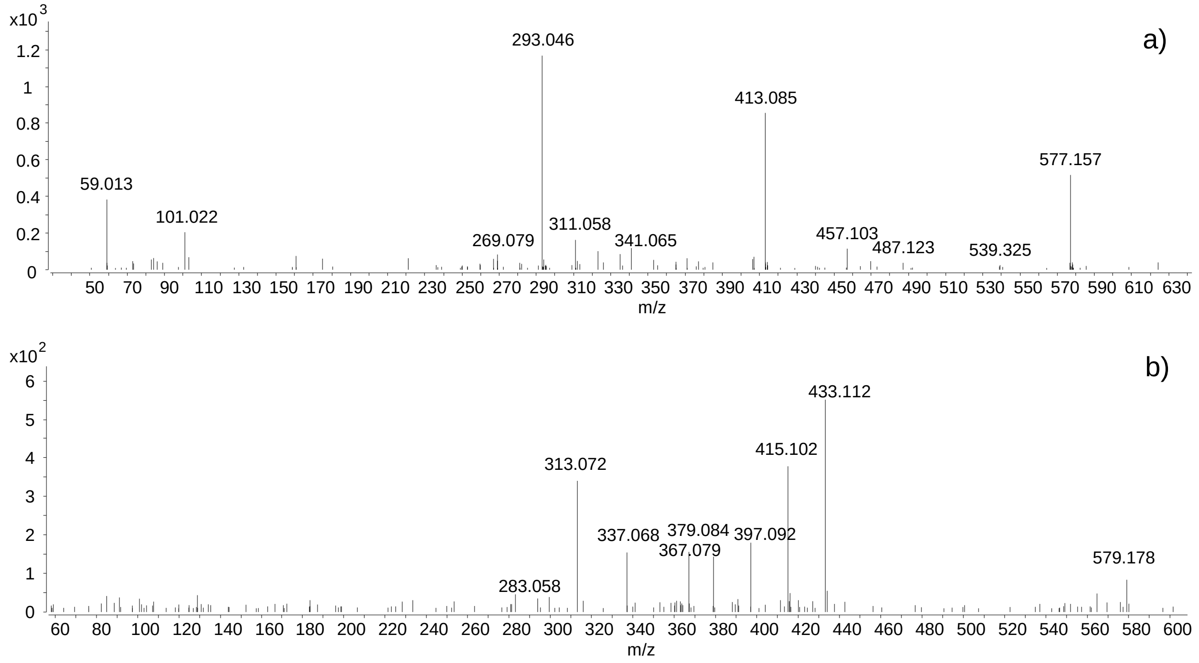

Figure S5. MS/MS tandem mass spectra of the unidentified molecule ($C_{27}H_{30}O_{14}$, MW = 578.164) obtained by HPLC-ESI-Q-ToF in a) negative and b) positive ionisation mode (Collision Induced Dissociation energy 30 V and 20 V respectively).

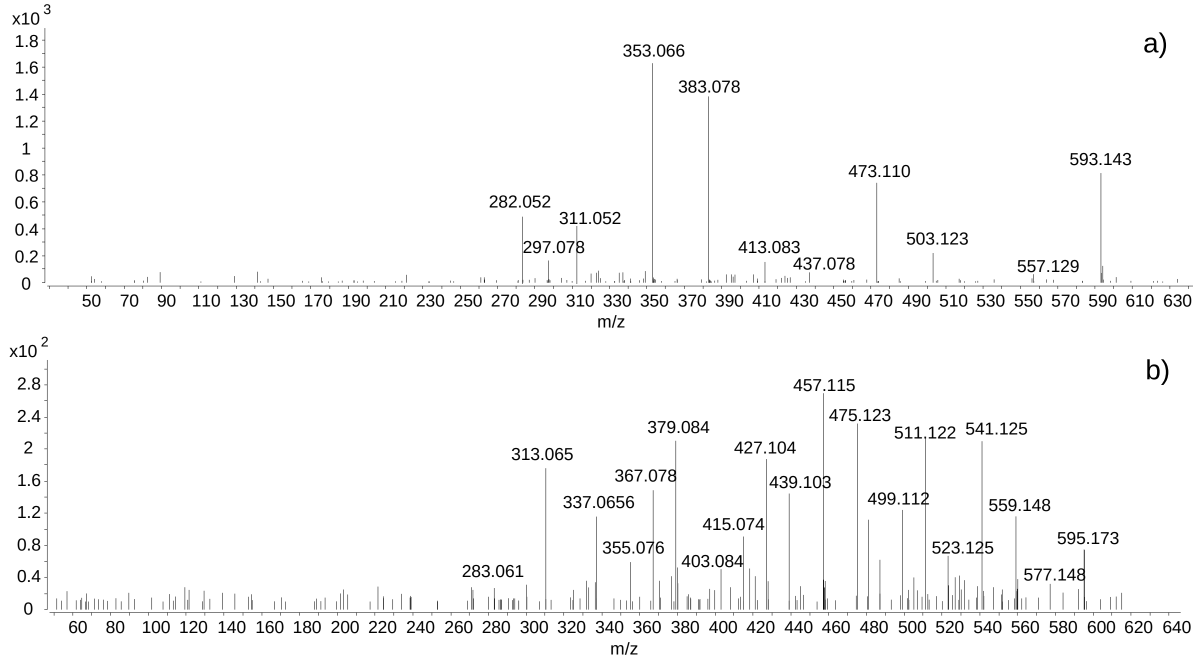

Figure S6. MS/MS tandem mass spectra of the unidentified molecule ($C_{27}H_{30}O_{15}$, MW = 594.159) obtained by HPLC-ESI-Q-ToF in a) negative and b) positive ionisation mode (Collision Induced Dissociation energy 35 V and 20 V respectively).

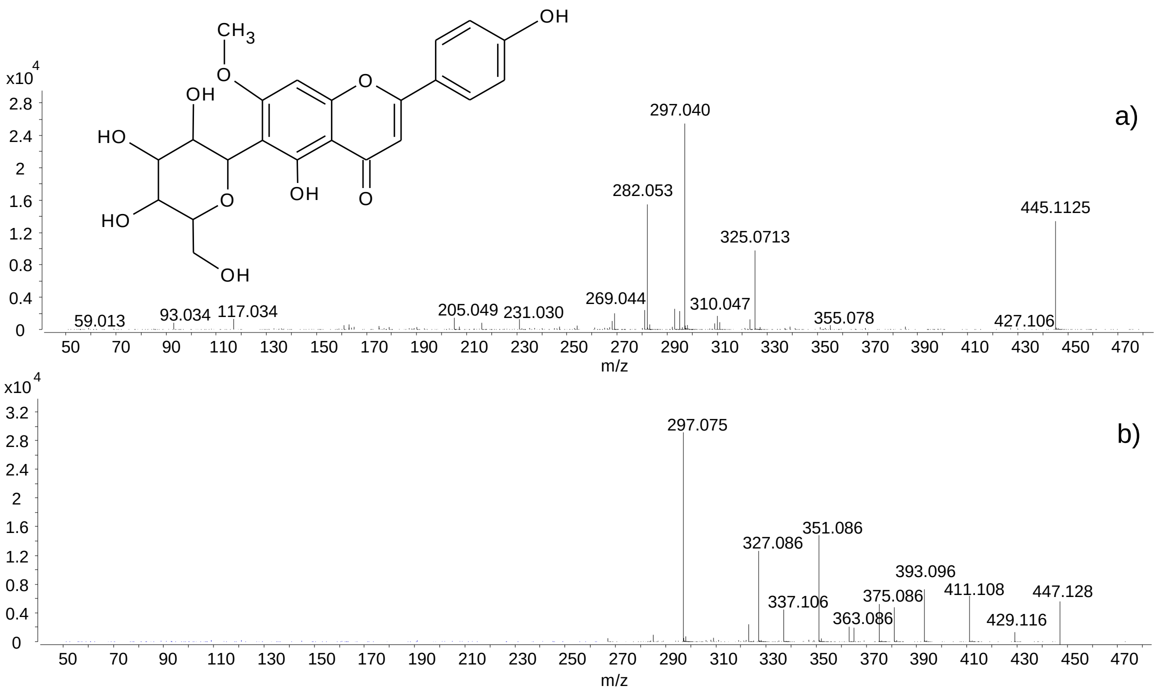

Figure S7. Molecular structure and MS/MS tandem mass spectra of swertisin obtained by HPLC-ESI-Q-ToF in a) negative and b) positive ionisation mode (Collision Induced Dissociation energy 35 V and 20 V respectively).

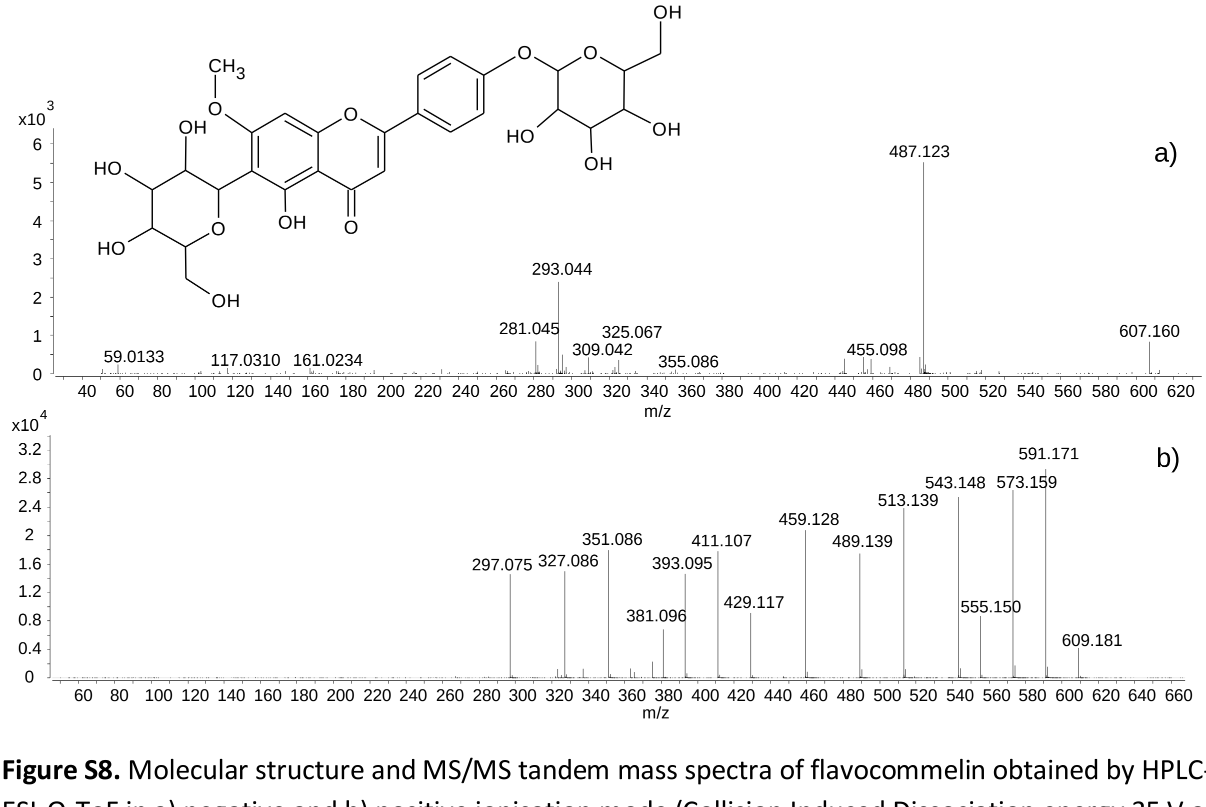

Figure S8. Molecular structure and MS/MS tandem mass spectra of flavocommelin obtained by HPLC-ESI-Q-ToF in a) negative and b) positive ionisation mode (Collision Induced Dissociation energy 35 V and 20 V respectively).

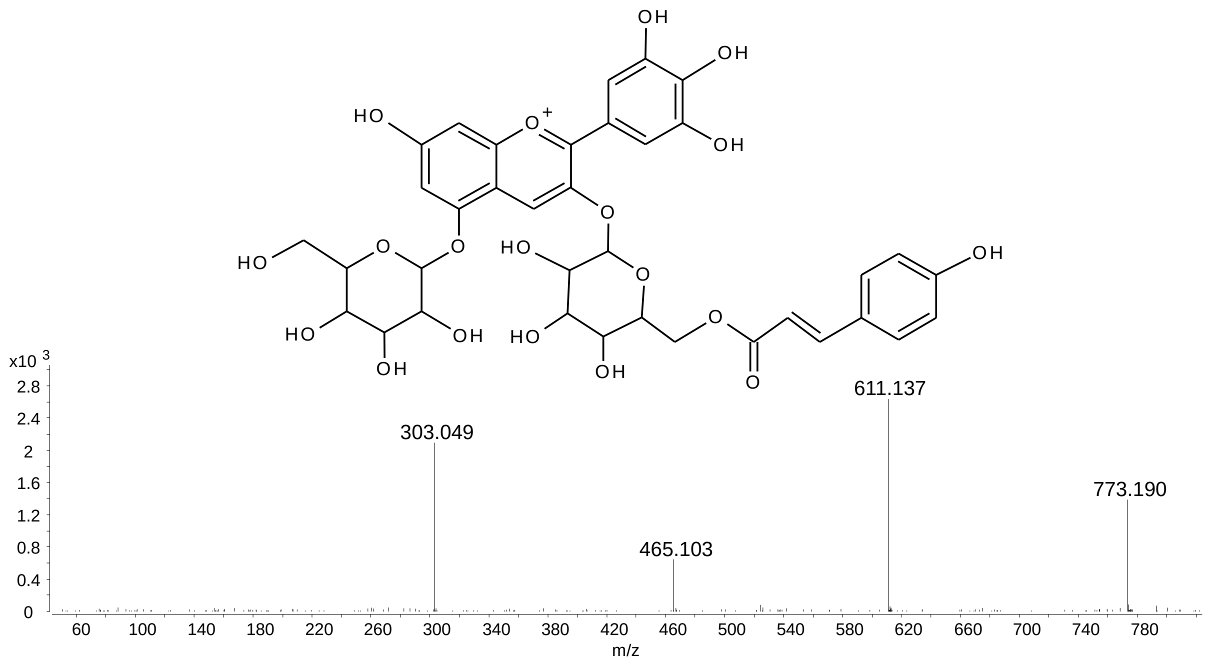

Figure S9. Molecular structure and MS/MS tandem mass spectrum of awobanin obtained by HPLC-ESI-Q-ToF in positive ionisation mode (Collision Induced Dissociation energy 20 V).

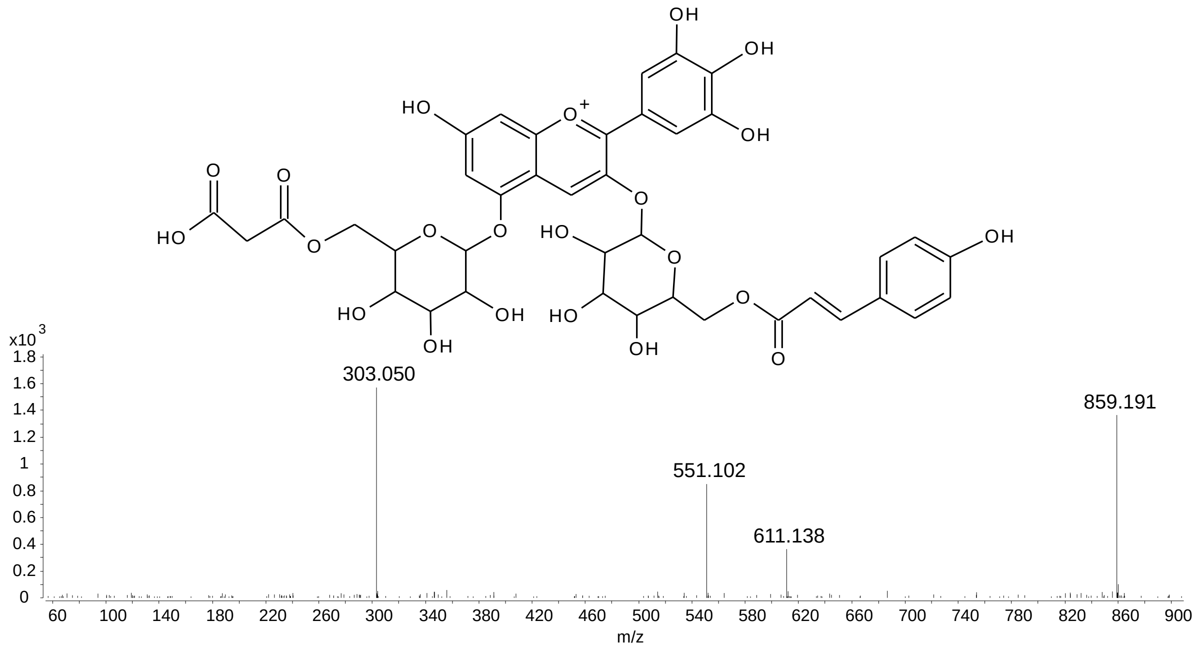

Figure S10. Molecular structure and MS/MS tandem mass spectrum of malonylawobanin obtained by HPLC-ESI-Q-ToF in positive ionisation mode (Collision Induced Dissociation energy 25 V).
